# Therapeutic plasma exchange in severe postpartum HELLP syndrome: a promising treatment

**DOI:** 10.1186/s12884-025-08585-x

**Published:** 2025-12-18

**Authors:** Onur Karaaslan, Gürcan Türkyılmaz, Latif Hacıoğlu, Çağrı Ateş, Erbil Karaman, Hanım Güler Şahin, Ali Doğan, Ersin Onat

**Affiliations:** 1 Department of Obstetrics and Gynaecology, Faculty of Medicine, Van Yüzüncü Yıl University, Van, 65090 Turkey; 2 Department of Obstetrics and Gynaecology, Faculty of Medicine, Lokman Hekim University, Ankara, Turkey; 3 Department of Haematology Van, Faculty of Medicine, Van Yüzüncü Yıl University, Van, Turkey

**Keywords:** Delivery, HELLP syndrome, Pregnancy, Therapeutic plasma exchange

## Abstract

**Objective:**

HELLP syndrome is a life-threatening obstetric complication marked by microangiopathic hemolytic anemia, elevated liver enzymes, and thrombocytopenia and potentially affecting multiple organ systems. While definitive treatment is typically achieved through delivery, in rare instances, both clinical and laboratory parameters may worsen in the postpartum period. In such critical cases, therapeutic plasma exchange (TPE) can serve as a potentially life-saving intervention.

**Methods:**

This study analyzed the laboratory and clinical responses of 21 patients diagnosed with severe HELLP syndrome who underwent postpartum TPE between 2010 and 2023.

**Results:**

Of the 21 patients, 14 (66.7%) were categorized as class 1 HELLP syndrome, and 7 (33.3%) as class 2. TPE was administered in a single session for 8 patients (38.1%), in two sessions for another 8 (38.1%), and in three sessions for the remaining 5 (23.8%). A significant increase in platelet count and significant decreases in lactate dehydrogenase (LDH), international normalized ratio (INR), activated partial thromboplastin time (APTT), alanine aminotransferase (ALT), aspartate aminotransferase (AST), and bilirubin levels were observed when compared before and after TPE. Despite intervention, severe complications—including sepsis, pulmonary edema, renal failure, cerebrovascular events, and disseminated intravascular coagulation (DIC)—developed in 9 patients (42.8%). One patient (4.8%) died due to multiorgan failure.

**Conclusion:**

These findings suggest that TPE administered in the postpartum period can significantly improve clinical outcomes, hematological and biochemical derangements in patients with severe HELLP syndrome. Early initiation of TPE, ideally within 24–48 h following delivery in patients unresponsive to supportive care, is strongly recommended.

**Supplementary Information:**

The online version contains supplementary material available at 10.1186/s12884-025-08585-x.

## Introduction

 Hemolysis, Elevated Liver enzyme levels, and Low Platelet levels (HELLP syndrome), first described by Weinstein in 1982[1], is a rare but severe pregnancy complication associated with high perinatal morbidity and mortality. The incidence of HELLP syndrome ranges from 0.2% to 0.8% in pregnancies, increasing to 10%–20% in women with preeclampsia [2–4]. The condition is marked by an excessive inflammatory response, activated coagulation and complement pathways, and elevated levels of endoglin, soluble fms-like tyrosine kinase-1 (sFlt1), tumor necrosis factor-alpha (TNF-α), and von Willebrand factor, all of which contribute to thrombotic microangiopathy (TMA) [5]. 

HELLP syndrome is commonly diagnosed using the Tennessee classification criteria [6, 7]. Prompt delivery is the primary treatment, with most cases recovering swiftly. However, in some instances, postpartum patients do not respond to supportive care, exhibiting rapid clinical and biochemical deterioration, including coagulopathy and multi-organ failure. In such cases, TMA plays a critical role in the pathophysiology, predominantly affecting the kidneys, brain, and liver. Given that HELLP syndrome is the most common pregnancy-associated TMA, recent studies have explored therapeutic plasma exchange (TPE) as a potential treatment for cases unresponsive to standard management [8, 9]. TPE facilitates the removal of pathogenic substances from the bloodstream, offering a potentially life-saving intervention.

This study aims to evaluate the clinical efficacy and laboratory impact of postpartum TPE in a cohort of patients with severe HELLP syndrome treated between 2010 and 2023.

## Methods

This retrospective study included 21 postpartum patients with severe HELLP syndrome who underwent TPE at the Faculty of Medicine, Van Yuzuncu Yil University, between 2010 and 2023. The study was approved by the local ethics committee and conducted in accordance with the Declaration of Helsinki. Written informed consent was obtained from all participants. Clinical data were retrieved from electronic medical records.

**Fig. 1:**
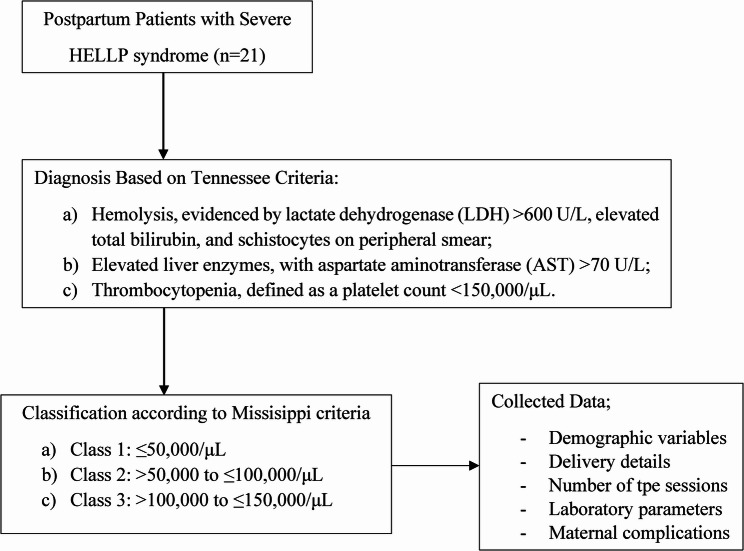
Flow diagram of patient selection and study inclusion

The diagnosis of HELLP syndrome was made based on the Tennessee criteria [6, 7], which require the presence of all three findings below and patients were divided into subgroups based on their platelet counts (PC) according to the Mississippi classification[10] (Fig. 1).

Seven patients met diagnostic criteria for disseminated intravascular coagulation (DIC), characterized by platelet count < 100,000/µL, fibrinogen < 300 mg/dL, D-dimer > 500 µg/L, and prolonged prothrombin time (PT ≥ 14 s) and activated partial thromboplastin time (APTT ≥ 40 s)[11]. Ten patients developed acute renal failure, defined by a creatinine clearance ≤ 20 mL/min. 

Data collected included demographic variables, delivery details, number of TPE sessions, laboratory parameters, and maternal complications. During postpartum follow-up, hemoglobin, platelets, LDH, INR, APTT, ALT, AST, Fibrinogen, Creatinine, and Total bilirubin levels were measured at 6-hour intervals. TPE was terminated when the platelet count exceeded 100,000, and the final values ​​were recorded. Differential diagnoses—such as thrombotic thrombocytopenic purpura (TTP), hemolytic uremic syndrome, acute fatty liver of pregnancy, and DIC secondary to massive hemorrhage—were systematically excluded based on clinical and laboratory findings. Although ADAMTS13 activity could not be measured due to technical limitations, TTP was ruled out clinically.

Oxygenation and ventilation, sedation, pain management, hemodynamic support, intensive monitoring, volume management, nutritional support, stress ulcer prevention, and venous thromboembolism prevention were applied to all patients as supportive therapy. TPE was initiated within 24–48 h postpartum for patients with multi-organ dysfunction unresponsive to supportive therapy. This decision was made collaboratively by a multidisciplinary team comprising obstetricians, hematologists, and intensive care specialists. When deciding on plasmapheresis, ASFA criteria were taken into consideration [12].

Procedures were performed using a Fresenius (AS104,AS204, Comtec) Multifiltrate Basic apheresis system. Gilcher’s method was used to determine the total blood volume (TBV), which allowed for the calculation of the total plasma volume (TPV) [TPV = TBV (1-hematocrit)] [13]. Each vial of 20% albumin (diluted till 5%) and each unit of FFP used as replacement fluids had a capacity of 50 mL and 250 mL, respectively. For vascular access, access and return lines are shown individually. The CVC was categorized as femoral, jugular, and subclavian. The fistula was classified as a peripheral vascular access channel group during statistical analysis, while tunneled catheters were classified as central venous catheters. The volume and number of replacement therapy were determined depending on the clinical characteristics of the patients. The course of treatment was maintained until either clinical stability or platelet counts above 100,000/µL.

Statistical analyses were conducted using Statistical Package for the Social Science System version 20.0. Continuous variables were expressed as mean ± standard deviation. Descriptive statistics were calculated, and dependent t tests were used to compare pre- and post-TPE parameters. A p-value < 0.05 was considered statistically significant.

## Results

The mean patient age was 32.8 ± 6.9 year. The mean gravidity and parity were 3.3 ± 1.5 and 2.7 ± 1.1, respectively. The mean body mass index was 27.5 ± 2.8, with most patients classified as overweight or obese. The mean gestational age at delivery was 32.2 ± 4.1 weeks. Nineteen (90.5%) patients underwent cesarean delivery.

Based on the Mississippi classification [10], fourteen (66.7%) patients were categorized as class 1, while the remaining seven (33.3%) cases were classified as class 2 and there are no class 3 patients. In class 3 patients, TPE is not usually needed because the platelet count is over 100,000. Only four (19%) patients had a history of preeclampsia, eclampsia, or HELLP syndrome in a prior pregnancy. Eight (38.1%) patients underwent a single TPE session, another eight (38.1%) received two sessions, and the remaining five (23.8%) required three sessions. Table 1 summarizes the vital signs, clinical and demographic characteristics of the patients.

**Table 1:** Demographic and clinical features of patients. *Bpm* Beats per minute

Patients’ characteristics (*n*:21)	mean±std or number of cases (%)
Age (years)	32.8±6.9
Gravidity	3.3±1.5
Parity	2.7±1.1
BMI (kg/m^2)^	27.5±2.8
Delivery weeks	32.2±4.1
Mississippi class	Class 1: 14 (66.7) Class 2: 7 (33.3) Class 3: 0
Preeclampsia/eclampsia history	Yes: 4 (%19) No 17 (%81)
Mode of delivery	Caesarean: 19 (90.5) Vaginal delivery: 2 (9.5)
TPE number	1: 8 (%38.1) 2: 8 (%38.1) 3: 5 (%23.8)
Vital Signs	Mean systolic arterial pressure: 163.5 (mmHg) Mean diastolic arterial pressure: 112.5 (mmHg) Mean Pulse: 92 (bpm) Mean Oxygen saturation (SPO_2)_: %94

All patients exhibited significant reductions in LDH, INR, APTT, ALT, AST, and total bilirubin levels. Conversely, platelet counts significantly increased following TPE therapy (*p* < 0.01). While hemoglobin levels showed an upward trend and creatinine levels decreased post-TPE, these changes were not statistically significant. Additionally, TPE had no significant impact on fibrinogen levels. Laboratory parameters before and after TPE treatment are presented in Table 2.

**Table 2:** Laboratory changes pattern before and after TPE (*n*:21) Haemoglobin, platelets, LDH, INR, Aptt, Alt, Ast, Fibrinogen, Creatinine, Total bilirubin

Laboratory parameters	Before TPE	After TPE	*p*
Haemoglobin (g/dl)	9.6 ±1.67	10.9 ±2.09	0.138
Platelets (μL)	35.952 ±25.765	208.3333 ±42.746	<0.001**
LDH (U/L)	2004 ±637	442 ±343	<0.001**
INR	1.15±0.3	0.75±0.12	<0.001**
APTT (seconds)	36.2 ± 16.9	18.8 ± 6.4	<0.001**
ALT (U/L)	305 ± 121	35 ± 13	<0.001**
AST (U/L)	393 ± 232	34 ± 8	<0.001**
Fibrinogen (mg/dl)	214.95 ± 97	246 ± 99.83	0.285
Creatinine (mg/dl)	1.47±0.67	1.08±0.62	0.052
Total bilirubin (mg/dl)	2.31±1.68	1.06±0.52	<0.001**

*P* values = <0.05, *p= <0.01, **p<0.001

Maternal mortality occurred in one (4.8%) patient in our cohort. This case, who delivered at 34 weeks of gestation and received two TPE sessions, died to sepsis secondary to a COVID-19 infection 10 days postpartum despite clinical and biochemical improvements. Pulmonary edema was observed in three patients (14.3%), all of whom were successfully treated with diuretics and fluid restriction. Renal insufficiency was identified in one (4.8%) patient, who subsequently required chronic dialysis. A cerebrovascular event occurred in one patient who was treated with thrombolytic therapy and recovered without severe sequelae. Three (14.3%) patients developed DIC and were successfully managed with blood products and fibrinogen. Maternal complications are detailed in Table 3.

**Table 3:** Maternal complications associated with HELLP syndrome

Complications	*N*: 21 (%)
Mortality	1 (4.8)
Sepsis	1 (4.8)
Pulmonary oedema	3 (14.3)
Renal insufficiency	1 (4.8)
Hepatic rupture/hematoma	0 (0)
Cerebrovascular insult	1 (4.8)
DIC	3 (14.3)

## Discussion

Our study suggests that TPE improves outcomes in patients with severe HELLP syndrome, reducing maternal morbidity and mortality. TPE facilitates a rapid increase in platelet counts while significantly lowering LDH, ALT, AST, INR, APTT, and bilirubin levels. We believe that TPE is a treatment method that should be kept in mind in severe cases where supportive treatment responses are present.

The precise etiology of HELLP syndrome remains unclear. Current hypotheses include fetal and maternal genetic mutations as well as inflammatory mechanisms. Recent studies emphasize immune maladaptation and placental-derived inflammatory cytokines as key contributors [14]. Delivery remains the definitive treatment for HELLP syndrome; however, a subset of patients may require intensive care due to continued deterioration postpartum. Agacayak et al.[15] evaluated 342 HELLP syndrome cases in a tertiary center, finding that 9.4% of patients required ICU admission. They reported significantly higher AST, LDH, bilirubin, and creatinine levels and significantly lower platelet counts in ICU-admitted patients.

HELLP syndrome is a classic form of TMA occurring during pregnancy and the postpartum period. Generalized endothelial dysfunction and TMA result from elevated circulating levels of trophoblast-derived antiangiogenic mediators, such as soluble endoglin and the soluble form of the vascular endothelial growth factor receptor (sFlt-1). Additionally, excessive complement activation has been observed in a subset of women with severe HELLP syndrome [16, 17]. TPE, an extracorporeal therapy, removes soluble components from plasma, targeting pathogenic substances while restoring essential plasma proteins. By eliminating anti-angiogenic factors and excessive complement activation, TPE can mitigate TMA and enhance recovery in HELLP patients [18]. According to ASFA guidelines, TPE is classified as a category 3 treatment for severe postpartum HELLP syndrome, indicating that its efficacy remains uncertain and should be considered on a case-by-case basis [19]. 

Several recent studies have explored TPE’s role in HELLP syndrome. Erkut et al.[20] evaluated 47 class 1 HELLP syndrome patients and observed significant clinical and laboratory improvements following TPE. They recommended postpartum TPE initiation within 24 h as an effective treatment option for class 1 HELLP syndrome. Chowdhry et al.[21] investigated nine patients who underwent TPE postdelivery for severe HELLP syndrome, demonstrating that early postpartum TPE improves clinical outcomes and resolves laboratory abnormalities. Simetka et al.[22] found that patients requiring TPE who failed to achieve clear improvements in platelet counts and AST levels within 48 h or those with acute kidney injury, neurological impairment, or respiratory distress syndrome—were at risk of progressing to TMA. They emphasized that TPE should be administered within 24–72 h postpartum. Komatsu et al.[23] conducted a nationwide survey in Japan advocating for early TPE therapy in severe HELLP syndrome cases associated with TMA.

Despite its widespread empirical use, corticosteroid therapy remains of uncertain efficacy in HELLP syndrome treatment. A Cochrane systematic review found no clear evidence supporting corticosteroids for improving substantive clinical outcomes, with their only benefit being an increase in platelet count [24]. Another systematic review by Wei-Jing Sun, encompassing seven randomized clinical trials, confirmed that corticosteroids did not provide significant clinical benefits for pregnant women with HELLP syndrome. These findings highlight the need for more definitive and effective treatment modalities in severe HELLP syndrome cases.

A major challenge in severe HELLP syndrome cases is differentiating it from other causes of TMA, including TTP, complement-mediated atypical hemolytic uremic syndrome (aHUS), and catastrophic antiphospholipid syndrome (cAPS). TTP is a rare, life-threatening hematologic disorder characterized by thrombocytopenia, TMA, and neurological involvement. aHUS, less common than TTP, presents with thrombocytopenia and severe renal failure. Differential diagnosis relies on thorough clinical evaluation and laboratory testing, particularly ADAMTS13 levels for TTP and complement abnormalities for aHUS. However, distinguishing these syndromes postpartum can be challenging due to overlapping clinical features. Moreover, most hospitals lack the capacity for rapid ADAMTS13 or complement testing, leading to diagnostic delays [25]. Nevertheless, as TPE is an effective treatment for all these conditions, an immediate differential diagnosis is not always essential [26]. 

### Limitations

This study has several limitations. First, its retrospective design and lack of a control group limit the strength of our conclusions. Additionally, we were unable to assess ADAMST13 and complement levels due to technical constraints. Despite these limitations, TPE has been described as a potential treatment for HELLP syndrome, although its efficacy remains uncertain and should be considered on an individual basis.

## Conclusion

HELLP syndrome remains a major contributor to maternal morbidity and mortality and requires prompt diagnosis and tailored management. Our results suggest that therapeutic plasma exchange is effective in correcting laboratory abnormalities and improving clinical outcomes, particularly when initiated within 24–48 h postpartum in class 1 and 2 HELLP syndrome cases. Further prospective, controlled studies are warranted to clarify the therapeutic role and optimal timing of TPE in this setting.

## Supplementary Information

Supplementary Material 1.

## Data Availability

The datasets generated and/or analysed during the current study are not publicly available due to patient confidentiality and the small number of cases, which may compromise anonymity. However, the data are available from the corresponding author on reasonable request.
